# Efficacy and Safety of Afatinib in the Treatment of Advanced Non-Small-Cell Lung Cancer with EGFR Mutations: A Meta-Analysis of Real-World Evidence

**DOI:** 10.1155/2021/8736288

**Published:** 2021-12-18

**Authors:** Lemeng Zhang, Yongzhong Luo, Jianhua Chen, Tianli Cheng, Hua Yang, Changqie Pan, Haitao Li, Zhou Jiang

**Affiliations:** Thoracic Medicine Department 1, Hunan Cancer Hospital, Changsha 410013, Hunan Province, China

## Abstract

**Introduction:**

The purpose of this study was to explore the efficacy and safety of afatinib in advanced non-small-cell lung cancer (NSCLC) patients with epidermal growth factor receptor (EGFR) mutations based on real-world evidence.

**Materials and Methods:**

Eligible real-world studies were identified from PubMed, Cochrane Library, and Embase. Cochrane guidelines were used to assess the quality of included studies. Cochran's *Q* test and I^2^ statistics were used for the heterogeneity analysis.

**Results:**

Twenty-five studies were included in this meta-analysis; nine studies were included in the qualitative descriptive analysis. The summarized disease control rate (DCR) was 87.6% (81.5%, 92.7%), and the overall response rate (ORR) was 58.9% (48.8%, 68.7%). The pooled median progression-free survival (PFS) was 12.4 (10.3, 14.5) months, mean time to failure (TTF) was 15.4 (13.6, 17.2) months, and median overall survival (OS) was 31.6 (26.7, 36.5) months. The total incidences of adverse events (AEs) for skin rashes, diarrhea, paronychia, and mucositis were 71.4% (64.4%, 77.9%), 70.4% (60.1%, 79.8%), 52.1% (41.9, 62.3%), and 36.5% (29.5%, 43.8%), respectively. The incidences of severe adverse events (SAEs, Grade ≥3) for diarrhea, skin rashes, paronychia, and mucositis were 9.7% (6.8%, 13.1%), 5.8% (4.5%, 7.2%), 3.8% (2.0%, 6.2%), and 2.1% (1.0%, 3.6%), respectively. Differences in PFS and OS between the afatinib non-full-dose (<40 mg) and full-dose (>40 mg) groups were not significant (*P* > 0.05). However, the ORR in the full-dose group was 78.5% (66.7%, 88.4%), which was significantly higher than that in the non-full-dose group (67.8% [56.8%, 77.9%]).

**Conclusion:**

The efficacy and safety of afatinib has been confirmed by real-world evidence in advanced NSCLC with EGFR mutation, consistent with randomized controlled trial results. In real-world setting, tolerability-guided dose adjustment might not affect the afatinib efficacy.

## 1. Introduction

Lung cancer is the leading cause of cancer-related deaths and a serious threat to human health [1]. Non-small-cell lung cancer (NSCLC) accounts for more than 80% of lung cancer [2]. Epidermal growth factor receptor (EGFR) mutations have been identified in approximately 50% of Asian and 10–15% of Caucasian lung adenocarcinoma patients [3, 4]. Currently, tyrosine kinase inhibitors (TKIs), including erlotinib, gefitinib, dacomitinib, afatinib, and osimertinib, are the standard first-line treatment for advanced NSCLC patients with EGFR mutations [5].

Afatinib is an irreversible second-generation ErbB family blocker [6], which has been approved as a first-line treatment for NSCLC patients with EGFR exon 19 deletions or exon 21 L858R substitution mutations [7]. In 2013, afatinib was approved worldwide as a first-line treatment for patients with EGFR-mutant NSCLC [5, 8]. The LUX-Lung 3/6/7 trials revealed that afatinib had obvious effects in the treatment of advanced EGFR-mutant NSCLC [9–12] and might provide a better curative effect than first-generation EGFR-TKIs [13]. Moreover, a meta-analysis based on randomized controlled trials (RCTs) has shown that afatinib prolonged progression-free survival (PFS), increased overall survival (OS), and the overall response rate (ORR) [14]. However, whether afatinib is effective in particular subgroups remains controversial due to the RCT exclusion criteria [14, 15]. Furthermore, the adverse effects of afatinib limit its clinical application [16]. Thus, a dose-adjustment strategy guided by tolerability can yield clinical benefits based on RCT data [14, 16], which need to be demonstrated through real-world evidence (RWE)-based data.

It is undeniable that the credibility of RCTs can be inferred from causality and is thus considered the gold standard of clinical research; however, RCTs may not reflect real-world practice due to strict inclusion criteria [17]. Patients in the real-world may differ in numerous characteristics from those in RCTs. RWE-based studies collect data of patients treated as a whole in clinical practice according to local government regulations, overcoming inherent limitations of RCTs, and can assess the efficacy and safety information of patients in the real world [18, 19]. Therefore, it is crucial to confirm the efficacy and safety of afatinib by using RWE data. Additionally, a previous study based on real-world data proved that afatinib dose adjustment decreases the intensity and frequency of adverse drug reactions without affecting the efficacy [20]. Although RWE contributes to the evaluation of certain clinical influential factors, the impact of dose adjustment, brain metastasis, mutation type, and Eastern Cooperative Oncology Group Performance Status (ECOG-PS) on the efficacy and safety of afatinib remains elusive.

Accordingly, we performed a meta-analysis based on real-world afatinib data in advanced NSCLC with EGFR mutations. To the best of our knowledge, this is the first meta-analysis based on RWE to explore the efficacy and safety of afatinib in advanced NSCLC with EGFR mutations. In this study, related RWE from Embase, PubMed, and Cochrane Library databases were analyzed. Herein, we aimed to comprehensively analyze the efficacy and safety of afatinib in NSCLC patients with advanced EGFR mutations based on real-world evidence. Furthermore, we explored the impact of tolerability-guided dose adjustment, brain metastasis, mutation type, and ECOG-PS.

## 2. Materials and Methods

This meta-analysis was performed following the guidelines of the meta-analyses (PRISMA) statement [21].

### 2.1. Data Sources

Relevant studies were searched and identified in electronic databases, including Embase, PubMed, and Cochrane Library (updated to December 31, 2020). The main search keywords included “afatinib,” “non-small cell lung cancer,” NSCLC, “lung adenocarcinoma,” and “adenosquamous carcinoma” The search was performed based on a combination of subject and free words. Additionally, a manual search of references in identified literature was performed to obtain additional information regarding the procedure. No language restrictions were applied to the current meta-analysis.

### 2.2. Inclusion and Exclusion Criteria

In the present study, the inclusion criteria employed were as follows: (i) the subjects were patients with advanced EGFR-mutated NSCLC diagnosed by histology and cytology; (ii) the study reported the efficacy (ORR, disease control rate (DCR), OS, PFS, and time to failure (TTF)) or safety (adverse reactions/serious adverse reactions) of afatinib in the treatment of NSCLC; (iii) the study reported the difference in efficacy and safety based on different groups, including different afatinib doses (full dose (40 mg/day for 6 months or more) *vs*. non-full dose (<40 mg/day for 6 months or 40 mg/day for a reduction in the first 6 months)), mutation type (exon 19 deletion *vs*. uncommon, exon 21 L858R *vs*. uncommon, exon 19 deletion *vs*. exon 21 L858R), ECOG-PS (0–1 *vs*. 2–4), and brain metastases; (iv) the research type was RWE.

The exclusion criteria were as follows: (i) nontreatment studies, including letters, reviews, and comments; (ii) the study lacked parameters required for quantitative analysis (including mean, sample size, standard deviation of the experimental group and the control group required for continuous variable meta-analysis) and could not be obtained through the conversion of other data in the study (enrolled as the qualitative description study); (iii) the study lacked baseline information such as gender, age, tumor classification, and ECOG-PS, reporting only the efficacy (PFS and TTF) of afatinib (enrolled as the qualitative description study); (iv) the research type was RCT. Furthermore, only one study (containing complete information) was extracted if more than one study was published using the same data.

### 2.3. Data Extraction and Quality Assessment

In the present study, two independent researchers participated in data extraction. The available data included first author name, year of publication, gender, sample size, history, country, age, stage, ORR, DCR, OS, PFS, TTF, and adverse reactions. The extraction tables were exchanged after both researchers had completed the above data extraction work. Any inconsistencies in extracted results were resolved through discussion.

### 2.4. Statistical Analysis

STATA software (version 11.0) was used for statistical analysis. The incidence rate (IR) and 95% confidence interval (CI) were used as the effect size to evaluate the incidence of ORR, DCR, AEs, and SAEs in patients with NSCLC administering afatinib. The median (95% CI) was used to assess the months of PFS, OS, and TTF, while weighted mean difference (WMD) and 95% CI were utilized as combined indices of effect quantity. The relative risk (RR) and 95% CI were used as the combined indices of effect quantity to comprehensively evaluate differences in ORR, DCR, and incidence of adverse reactions between the two groups. Moreover, the risk of OS/PFS was analyzed using hazard risk (HR) and 95% CI. If the study did not directly report the HR (95% CI) of OS and PFS but reported the median survival and log-rank *P*-value in non-full dose *vs*. full dose of afatinib, the HR (95% CI) could be converted using the method described before [22].

Cochran's *Q* test and *I*^2^ statistics were used to perform the heterogeneity analysis [23]. A random-effects model was used if heterogeneity was obtained (*P* < 0.05, *I*^2^ > 50%); conversely, a fixed-effects model was employed. Moreover, to assess the effect of the above factors on the combined results of PFS, a subgroup analysis was conducted on PFS based on the timing of afatinib administration and data sources (direct reports in study or obtained by conversion). Furthermore, a sensitivity analysis was undertaken to assess the effect of combined results by analyzing the relevant studies individually. Finally, the Egger test [24] was used to analyze publication bias between the two groups.

## 3. Results

### 3.1. Literature Review and Characteristics of Included Studies

**Figure 1:**
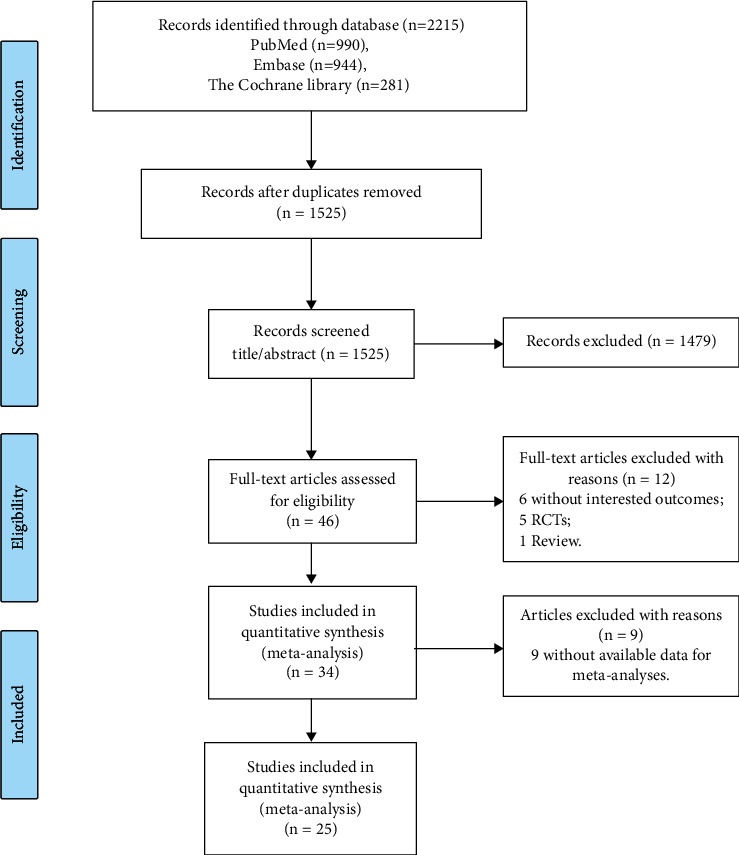
Flow diagram of the screening process for eligible studies.

In total, 994, 990, and 281 studies were explored in the Embase, PubMed, and Cochrane Library databases, respectively (Figure 1). After eliminating duplicate studies, a total of 1525 studies were obtained, with 1479 studies further excluded as these failed to meet the inclusion criteria after assessing the abstract and title. Among the remaining 46 studies, 12 relevant studies were identified after reading the full text. Manual retrieval did not detect any study that could be included in the current analysis. Finally, a total of 25 studies [20, 25–33] with sufficient data were included (Table 1). A total of nine studies [34–41] were included in the current qualitative descriptive analysis (Table 2).

### 3.2. Study Characteristics

The 25 enrolled studies were published between 2014 and 2020. The study areas were primarily in Asia. Moreover, 19, 1, and 5 studies were enrolled in first-line, second-line, and mixed population groups, respectively.

### 3.3. Meta-Analysis for Efficacy and Safety of Afatinib Based on RWE

**Figure 2:**
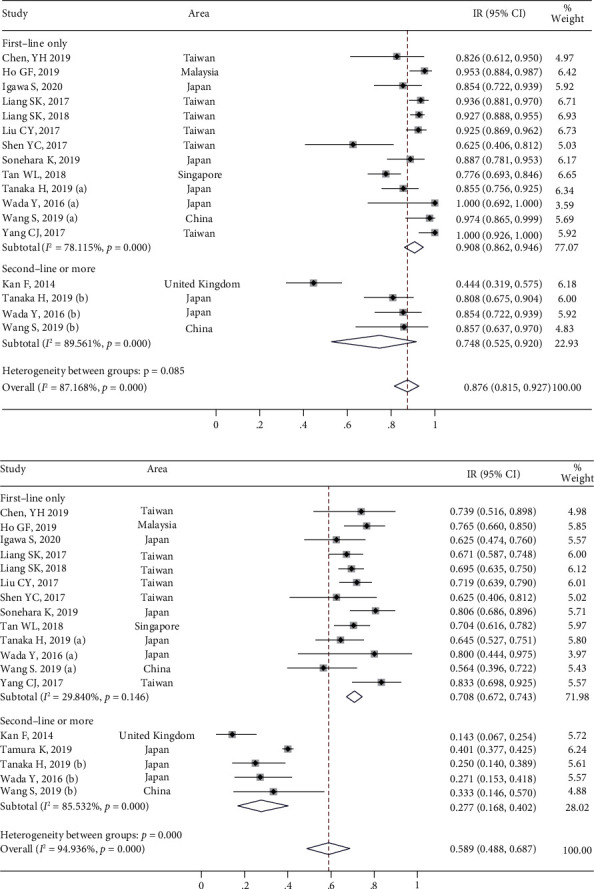
The meta-analysis results of afatinib efficacy and safety in advanced NSCLC with EGFR mutation: (a) the outcome of disease control rates (DCRs) and (b) the outcome of objective response rates (ORRs).

A total of 15 studies presented the outcomes of DCR (Figure 2(a)) and ORR (Figure 2(b)). Among these 15 studies, the heterogeneity was statistically significant (*I*^2^ > 50%, *P* < 0.001), and the combined result of DCR was 87.6% (95% CI [81.5%, 92.7%]), and the difference between the first-line-only group (90.8%; 95% CI [86.2%, 94.6%]) and second-line group (74.8%; 95% CI [52.5%, 92.0%]) was not significant (*P* > 0.05). The combined ORR was 58.9% (95% CI [48.8%, 68.7%]). The difference in ORR between the first-line-only group (70.8%; 95% CI [67.2%, 74.3%]) and second-line group (22.7%; 95% CI [16.8%, 40.2%]) was significant (*P* < 0.05).

**Figure 3:**
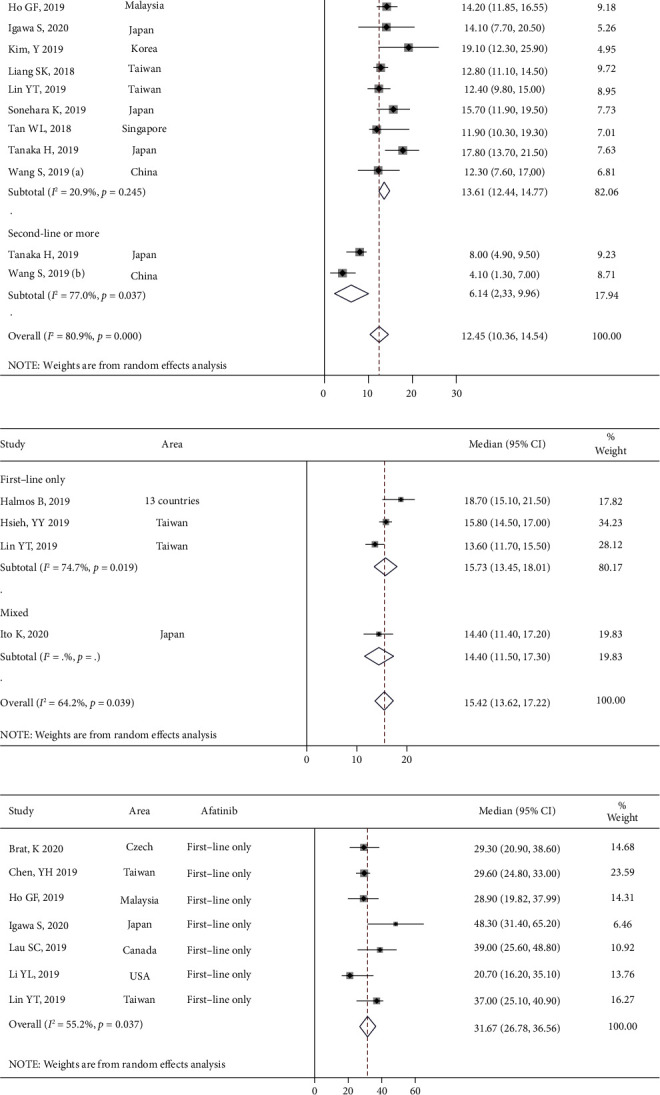
The meta-analysis results of afatinib efficacy and safety in advanced NSCLC with EGFR mutation: (a) the outcome of progression-free survival (PFS); (b) the outcome for overall survival (OS); and (c) the outcome for time to failure (TTF).

Moreover, a total of 12 studies indicated the PFS outcomes of afatinib (Figure 3(a)), and the heterogeneity was statistically significant (*I*^2^ > 50%, *P* < 0.05). The combined median PFS was 12.4 months (95% CI [10.3, 14.5]). For PFS, the difference between the first-line-only group (13.6 months; 95% CI [12.4, 14.7]) and second-line group (6.1 months; 95% CI [2.3, 9.9]) was significant (*P* < 0.05). Furthermore, four studies reported TTF outcomes (Figure 3(b)). The heterogeneity was statistically significant (*I*^2^ > 50%, *P* < 0.05), and the combined median TTF was 15.4 months (95% CI [13.6–17.2]). The difference in TTF between the first-line-only group (15.7 months, 95% CI [13.4, 18.0]) and the mixed group (14.4 months, 95% CI [11.5, 17.3]) was not significant (*P* > 0.05). Seven studies reported the OS outcomes in patients treated with afatinib (Figure 3(c)). The heterogeneity test among these seven studies was statistically significant (*I*^2^ > 50%, *P* < 0.05). The combined median OS was 31.6 months (95% CI [26.7–36.5]).

**Figure 4:**
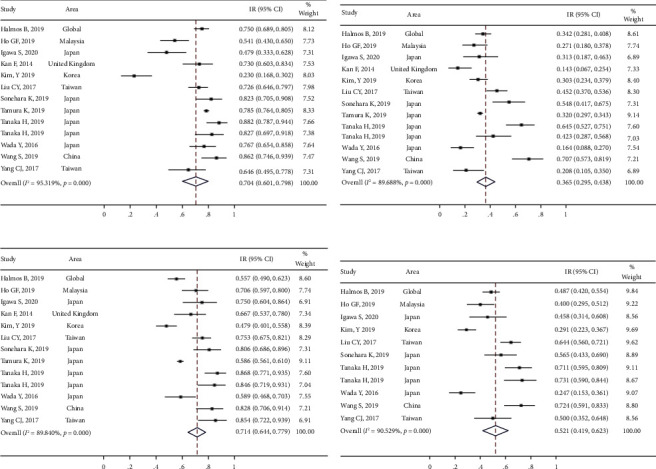
The meta-analysis results for various incidence rates after afatinib treatment in advanced NSCLC with EGFR mutation: (a) the incidence rate of diarrhea; (b) the incidence rate of mucositis; (c) the incidence rate of skin rashes; and (d) the incidence rate of paronychia.

**Figure 5:**
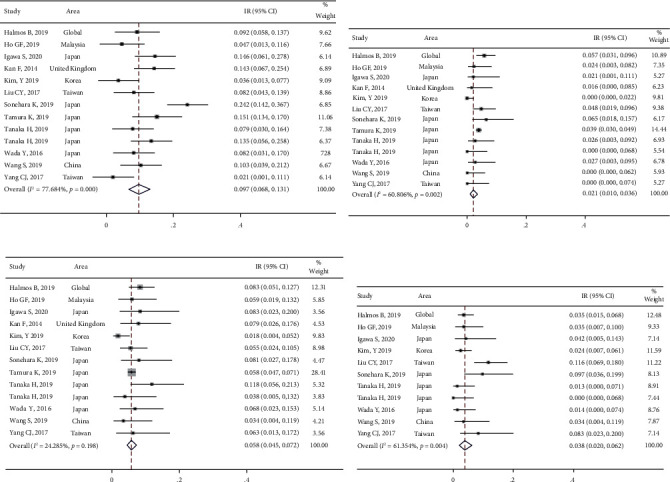
The meta-analysis results for serious adverse reaction incidence rates after afatinib treatment in advanced NSCLC with EGFR mutation: (a) the adverse reaction incidence rate of diarrhea; (b) the adverse reaction incidence rate of mucositis; (c) the adverse reaction incidence rate of skin rashes; and (d) the adverse reaction incidence rate of paronychia.

In total, 12 studies reported the incidence of diarrhea, mucositis, and skin rashes and 10 studies reported the total incidence of paronychia, including any grade (Figures 4(a)–4(d)) and Grade ≥3 (Figures 5(a)–5(d)), respectively. The results revealed that the most common AEs were diarrhea, mucositis, skin rashes, and paronychia, with an incidence of 70.4% (95% CI [60.1%, 79.8%]) (Figure 4(a)), 36.5% (95% CI [29.5%, 43.8%]) (Figure 4(b)), 71.4% (95% CI [64.4%, 77.9%]) (Figure 4(c)), and 52.1% (95% CI [41.9%, 62.3%]) (Figure 4(d)), respectively. Meanwhile, the incidences of common SAEs (Grade ≥3) for diarrhea, mucositis, skin rashes, and paronychia were 9.7% (95% CI [6.8%, 13.1%]) (Figure 5(a)), 2.1% (95% CI [1.0%, 3.6%]) (Figure 5(b)), 5.8% (95% CI [4.5%, 7.2%]) (Figure 5(c)), and 3.8% (95% CI [2.0%, 6.2%]) (Figure 5(d)), respectively.

Additionally, the total incidence of fatigue was 15.1% (95% CI [4.1, 30.9], Supplementary Figure 1A) and the incidence of severe fatigue was 0.8% (95% CI [0.0, 2.2], Supplementary Figure 2A). The total and severe alanine aminotransferase (ALT) levels elevated were 8.3% (95% CI [0.0%, 31.0%]) (Supplementary Figure 1B) and 0.7% (95% CI [0.0%, 2.3%]) (Supplementary Figure 2B), respectively. The incidence of total and severe aspartate aminotransferase (AST) levels elevated was 6.2% (95% CI [0.0%, 34.3%]) (Supplementary Figure 1C) and 0.3% (95% CI [0.0%, 1.5%]) (Supplementary Figure 2C), respectively. The incidence of total and severe interstitial lung disease (ILD) was 1.1% (95% CI [0.1%, 2.9%]) (Supplementary Figure 1D) and 0.7% (95% CI [0.0%, 2.3%]) (Supplementary Figure 2D), respectively. Finally, except for skin rashes and fatigue, significant heterogeneity was observed among all studies that contained other indicators (*I*^2^ > 50%, *P* < 0.05).

### 3.4. Effect of Tolerability-Guided Afatinib Dose Adjustment

**Figure 6:**
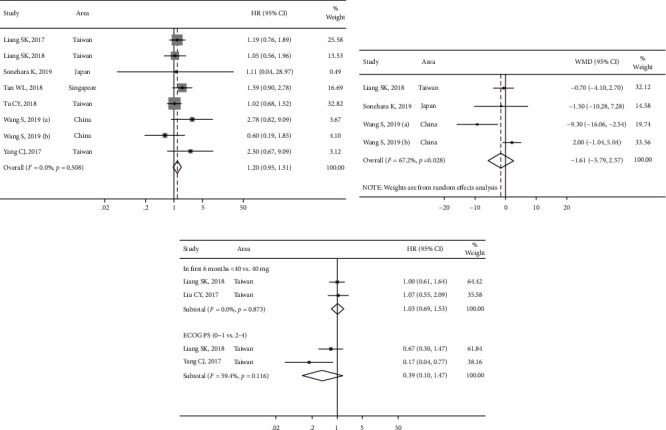
The meta-analysis results for progression-free survival (PFS) among groups after afatinib treatment in advanced NSCLC with EGFR mutation: (a) PFS between non-full-dose group and full-dose group; (b) PFS between the full-dose group and non-full-dose group; and (c) the OS between non-full-dose group and full-dose group in advanced NSCLC patients with epidermal growth factor receptor (EGFR) positive mutations after afatinib treatment.

Compared with the full dose of 40 mg/day, 57.8% (1917/3319) patients administered non-full dose of afatinib (Table 1). Seven studies (8 sets of research data) reported PFS between the non-full-dose and full-dose groups (Figure 6(a)). Among them, HRs (95% CI) in four studies (five sets of data) were obtained by conversion. The heterogeneity for these data was not significant (*I*^2^ < 0.0%, *P* > 0.05). For the combined results, the HR was 1.2 (95% CI [0.9, 1.5]; *P* > 0.05) (Table 3, Figure 6(a)), which indicated that dose reduction does not impact the therapeutic efficacy in terms of PFS. The PFS of the non-full-dose group was 5.0–14.2 months, while the PFS in the full-dose group was 3.0–15.7 months. For the combined results, the WMD was −1.6 months (95% CI [−5.7, 2.5]) (Figure 6(b)). Two studies reported the difference in OS risk between the non-full-dose and full-dose groups (Figure 6(c)). The result of the heterogeneity test indicated that the heterogeneity between the two groups was not significant (*I*^2^ < 0.0%, *P* > 0.05); thus, for the combined result, the HR was 1.03 (95% CI [0.69, 1.5]; *P* > 0.05), which indicated that dose reduction did not affect the therapeutic efficacy in terms of OS.

**Figure 7:**
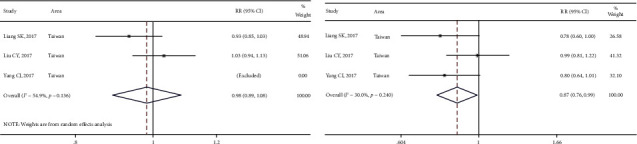
The meta-analysis results for disease control rates (DCRs) and objective response rates (ORRs) in different dose groups after afatinib treatment in advanced NSCLC with EGFR mutation: (a) comparison of DCRs in different dose groups and (b) comparison of ORRs in different dose groups.

Three studies reported the difference between DCR and ORR based on different afatinib doses. The heterogeneity of DCR in these studies was significant (*I*^2^ > 50%, *P* > 0.05). The non-full-dose group (95.1% [88.0%, 99.4%]) presented a DCR similar to the full-dose group [95.6% (90.3, 99.1%)]. For the combined results, the RR was 0.9 (95% CI [0.8, 1.0]; *P* > 0.05) (Figure 7(a)). Moreover, the results of the heterogeneity test for ORR were *I*^2^ < 50% and *P* > 0.05. The non-full-dose group (67.8 [56.8%, 77.9%]) presented a significantly lower ORR than the full-dose group (78.5 [66.7%, 88.4%]). For the combined results, the RR was 0.8 (95% CI [0.7, 0.9]; *P* > 0.05) (Figure 7(b)). Although the difference in PFS and OS was not significant, the ORR was significantly higher in the full-dose group compared with the non-full-dose group.

**Figure 8:**
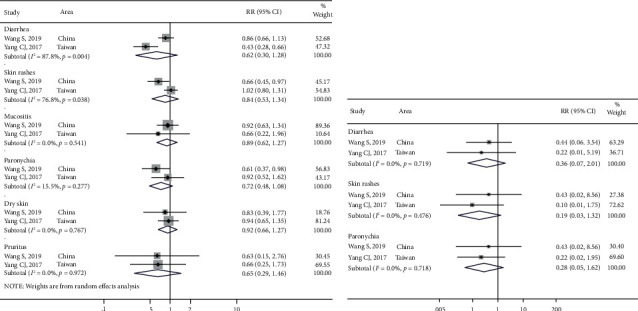
The meta-analysis results for total adverse events and severe adverse events in different dose groups after afatinib treatment in advanced NSCLC with EGFR mutation: (a) comparison of total adverse events in different dose groups and (b) comparison of severe adverse events in different dose groups.

Two studies reported differences in total AEs based on the full-dose and non-full-dose groups. Significant heterogeneity in the total AEs was observed in diarrhea and skin rashes (*I*^2^ > 50.0%; *P* < 0.05) but was not observed in mucositis and paronychia (*I*^2^ > 50.0%, *P* < 0.05) (Figure 8(a)). The heterogeneity of diarrhea, skin rashes, and paronychia (Grade ≥3) was not significant (*I*^2^ > 50.0%, *P* < 0.05) (Figure 8(b)). The incidence of total AEs and SAEs, including diarrhea, skin rash, mucositis, paronychia, dry skin, and pruritus, showed a decreased trend in the non-full-dose group (Table 4), which indicated the tolerability-guided dose adjustment alleviated afatinib-related adverse effects.

### 3.5. Effect of Subgroup on Efficacy and Safety of Afatinib

**Figure 9:**
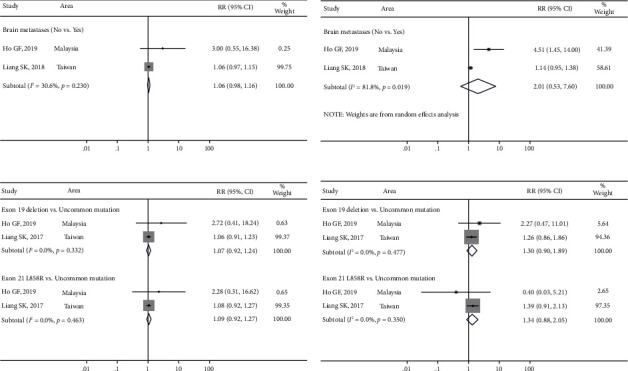
The meta-analysis results for disease control rates (DCRs) and objective response rates (ORRs) in subgroups after afatinib treatment in advanced NSCLC with EGFR mutation: (a) comparison of DCRs in different status of brain metastases; (b) comparison of ORRs in different status of brain metastases; (c) comparison of DCRs in different mutation sites; and (d) comparison of ORRs in different mutation sites.

Two studies reported differences in the DCR and ORR in brain metastases. The DCRs in the without metastases group and the brain metastases group were 94.6% (91.3%, 97.3%) and 89.1% (82.2%, 94.6%), respectively. The ORRs in the without metastases group and the brain metastases group were 74.9% (95% CI [69.1%, 80.2%]) and 61.8% (95% CI [52.2%, 71.0%]), respectively. The differences in DCR (RR: 1.0, 95% CI [0.9, 1.1]; *P* > 0.05) and ORR (RR: 2.0, 95% CI [0.5, 7.6]; *P* > 0.05) (Figures 9(a) and 9(b)) were not significant.

Furthermore, two studies reported differences in DCR/ORR between mutation types. The DCRs in the exon 19 deletion group, exon 21 L858R group, and uncommon group were 94.0% (95% CI [89.4, 97.4%]), 92.7% (95% CI [80.5%, 99.7%]), and 92.8% (95% CI [97.2%, 100.0%]), respectively. The differences in DCR in the exon 19 deletion *vs*. uncommon group (RR: 1.1, 95% CI [0.9, 1.2]; *P* > 0.05) and exon 21 L858R *vs*. uncommon group (RR: 1.1, 95% CI [0.9, 1.3]; *P* > 0.05) were not significant (Figure 9(c)). The ORRs in the exon 19 deletion group, exon 21 L858R group, and uncommon group were 71.9% (95% CI [64.3%, 78.9%]), 69.1% (95% CI [55.2%, 84.0%]), and 59.9% (95% CI [41.3%, 77.4%]), respectively. The differences in ORR in the exon 19 deletion *vs*. uncommon group (RR: 1.3, 95% CI [0.9, 1.9], *P* > 0.05) and exon 21 L858R *vs*. uncommon group (RR: 1.3, 95% CI [0.9, 2.0]; *P* > 0.05) were not significant (Figure 9(d)).

Moreover, two studies reported PFS in patients with brain metastases based on the full-dose and non-full-dose groups (Supplementary Figure 3A). For the combined results, the HR was 2.4 (95% CI [0.9, 5.9]; *P* > 0.05). Furthermore, the combined results showed that the PFS in the exon 19 deletion group was undoubtedly higher than that in the uncommon mutation group (HR: 0.2, 95% CI [0.1, 0.4]; *P* < 0.05), and PFS in patients without brain metastasis was significantly lower than that in patients with brain metastasis (HR: 0.5; 95% CI [0.4, 0.8]; *P* < 0.05) (Supplementary Figure 3B). No significant difference was observed in ECOG-PS (0–1) *vs*. ECOG-PS (≥2) (HR: 0.3, 95% CI [0.1, 1.4]; *P* > 0.05) (Supplementary Figure 3B). However, the heterogeneity between the two studies was significant (*I*^2^ = 59.4%) (Figure 6(c)).

### 3.6. Sensitivity Analysis and Publication Bias

Publication bias and sensitivity analyses were performed on the study outcomes. The sensitivity analysis results revealed that none of the included studies had a noticeable influence on the combined result of PFS between the full-dose and the non-full-dose groups. The combined results of PFS ranged from HR: 1.1 (95% CI [0.8, 1.4]) to HR: 1.3 (95% CI [0.9, 1.7]) (*P* > 0.05) following removal of any single study. Furthermore, the Egger test showed that publication bias in the current meta-analysis was not significant (*P* > 0.05).

## 4. Discussion

Based on RCT data, a meta-analysis has revealed that in the first-line treatment of EGFR-mutated NSCLC, there is no conclusive evidence that afatinib is more effective than gefitinib or erlotinib [15]. Meanwhile, Wang et al. have performed a meta-analysis of RCTs in advanced NSCLC to assess the safety and efficacy of afatinib when compared with chemotherapy and first-generation EGFR-TKIs. Their results revealed that compared with control groups, afatinib treatment apparently increased ORR (RR: 1.8, 95% CI [1.1–2.9]) and improved PFS (HR: 0.5; 95% CI) and improved OS (HR: 0.9, 95% CI [0.8–0.9]). In terms of safety, the incidence of adverse events (Grade ≥3) was as follows: diarrhea (11.8%) (RR: 8.9, 95% CI [5.3–14.9]), stomatitis (4.8%) (RR: 6.4, 95% CI [1.2–32.7]), and skin rash (10.7%) (RR: 7.3, 95% CI [1.5–34.1]) [14]. In this RWE-based meta-analysis, the results confirmed that the afatinib was with ORR 58.9% (48.8, 68.7), PFS 12.4 months (10.3, 14.5), TTF 15.4 months (13.6, 17.2), and OS 31.6 months (26.7, 36.5), which is consistent with RCT results. The incidences of severe adverse events (Grade ≥3, SAEs) for diarrhea, skin rashes, paronychia, and mucositis were 9.7% (6.8%, 13.1%), 5.8% (4.5%, 7.2%), 3.8% (2.0%, 6.2%), and 2.1% (1.0%, 3.6%), respectively. Furthermore, in the present study, the efficacy of afatinib in the first-line-only group was significantly superior to that in the second-line treatment. Therefore, the efficacy and safety of afatinib has been confirmed by RWE.

The efficacy of tolerability-guided dose adjustment remains controversial. Previously, it has been suggested that 40 mg was the recommended afatinib dose for first-line therapy [10]. A recent study has revealed that the PFS of the non-full-dose group was 12.8 months, while the PFS was 11.0 months for the full-dose group; however, the difference was not significant (HR: 1.3, 95% CI [0.9–2.0]) [42]. Yang et al. have reported that afatinib 30 mg daily as an initial dose presents a similar response rate and PFS as an initial dose of 40 mg daily [43]. In the current RWE-based meta-analysis, the results revealed that the difference in PFS and OS between the afatinib non-full-dose group (<40 mg) and full-dose group (>40 mg) was not significant (*P* > 0.05). However, the ORR in the full-dose group was 78.5% (95% CI [66.7%, 88.4%]), which was significantly higher than that in the non-full-dose group (67.8%; 95% CI [56.8, 77.9]). Thus, the real-world data suggested that decreasing the afatinib dose does not negatively impact efficacy; the full dose should be employed for treating NSCLC patients with EGFR mutations if tolerance permits.

Furthermore, moderate-to-severe adverse drug reactions usually result in dose reduction or discontinuation. Numerous clinical trials have reported that afatinib 40 mg daily as the starting dose presented severe adverse drug reactions, including skin rash, paronychia, and diarrhea [11, 12, 44]. 40 mg afatinib daily presented a significantly higher incidence of Grade 3 skin rash (16% *vs*. 0%) and diarrhea (100% *vs*. 41%) than 30 mg daily afatinib [45]. In the present study, the frequency and severity of adverse events (including diarrhea, skin rash, mucositis, paronychia, and pruritus) was higher in patients who administered 40 mg afatinib daily than in those who administered 30 mg afatinib daily. However, the differences of adverse reactions in the two groups of tolerability-guided afatinib dose adjustment were not significant. Moreover, compared with the 40 mg/day dose, 57.8% (1917/3319) of patients received a lower afatinib dose, with only 0.5% (18/3319) of patients receiving a higher afatinib dose; this could partly explain the lower tolerability and higher toxicities associated with afatinib 40 mg daily. However, it should be noted that the anticancer efficacy ORR of afatinib 30 mg daily did not surpass that of the 40 mg daily dose. Besides, the incidence and severity of adverse reactions showed a decreased trend in patients receiving non-full dose, which indicated the tolerability-guided dose adjustment alleviated afatinib-related adverse effects. Thus, the real-world data support that dose adjustment can be guided according to tolerance once adverse reactions occur.

In addition to the afatinib dose, clinical factors such as brain metastases can influence the results of patients with advanced EGFR-mutant NSCLC [46]. In the LUX-Lung 6 trial, the median PFS of patients with brain metastases treated with afatinib was lower than that of patients without brain metastases [47], which was in accordance with our results, suggesting that brain metastases is an influence factor of patients with advanced EGFR-mutant NSCLC based on afatinib dose.

The current study was the first RWE-based meta-analysis to explore the efficacy and adverse reactions in patients with advanced EGFR-mutated NSCLC. However, some limitations persist in the current study: (1) the small sample size of some included studies influenced certain outcome indicators of meta-analysis; (2) it was not possible to assess the methodological quality of included studies and the impact of quality on the results in this RWE study owing to a lack of suitable quality evaluation tools; (3) subgroup analysis was not performed on first-generation or second-generation EGFR-TKIs for comparing afatinib with erlotinib, dacomitinib, and gefitinib.

In conclusion, afatinib is a safe and effective first-line treatment in patients with EGFR-mutated NSCLC, and tolerability-guided afatinib dose adjustment might not affect the PFS of these patients. This study was performed based on real-world data, reflecting information on curative effects in real-world patients and fully compensates for disadvantages of RCTs.

## Figures and Tables

**Table 1:** Characteristics of 25 included studies in this meta-analysis.

Study	Area	Afatinib	N	Female, *n* (%)	Age, years, median (range)	Mutations	Clinical stage	ECOG-PS, 0–1/≧2	Histology	Dose reduce, no/yes
Brat, K 2020	Czech	First-line only	147	96 (65.3)	62.8 ± 11.1	EGFR mutation	12 IIIB, 135 IV	147/0	145 AD, 2 other	NR/NR
Chen YH, 2019	Taiwan	First-line only	23	15 (65.2)	42–81	17 Del19, 4 L858R, 2 uncommon	23 advanced	NR	NR	NR/NR
del Re M, 2019	Italy	First-line only	41	20 (48.8)	70.5 ± 11.3	34 Del19, 7 L858R	2 IIIB, 39 IV	NR	NR	26/15
Halmos B, 2019	Global	First-line only	228	138 (60.5)	67.0 (32.0–90.0)	178 Del19, 49 L858R	12 IIIB, 216 IV	192/27 (9 missing)	226 AD, 1 SCC, 1 NOS	51/173
Ho GF, 2019	Malaysia	First-line only	85	47 (55.3)	59.1 ± 10.8	68 Del19, 11 L858R, 6 uncommon	4 IIIB, 81 IV	69/16	82 AD, 3 SCC	49/26
Hsieh YY, 2019	Taiwan	First-line only	751	424 (56.5)	62.5 ± 11.2	EGFR mutation	43 IIIB, 708 IV	678/73	735 AD, 16 other	NR/NR
Igawa S, 2020	Japan	First-line only	48	27 (56)	67 (35–85)	29 Del19, 19 L858R	36 IV, 12 recurrence	24/24	NR	21/27
Ito K, 2020	Japan	Mixed	218	102 (46.8)	64.3 (34–87)	141 Del19, 47 L858R, 30 uncommon	39 IA-IIIA, 11 IIIB, 168 IV	203/12 (3 missing)	208 AD, 10 non-AD	NR/NR
Kan F, 2014	United Kingdom	Second-line or more	63	37 (58.7)	64 (29–83)	15 Del19, 13 L858R, 4 uncommon	63 IV	50/12 (1 missing)	58 AD, 1 SCC, 2 NOS, 1 large cell, 1 unknown	33/31
Kim, Y 2019	Korea	First-line only	165	80 (48.5)	57 (30–79)	114 Del19, 37 L858R, 14 uncommon	165 IV	156/9	NR	53/112
Lau SC, 2019	Canada	First-line only	70	44 (63)	62 (34–84)	41 Del19, 20 L858R, 9 uncommon	70 advanced	68/2	66 AD, 4 other	NR/NR
Li YL, 2019	USA	First-line only	87	62 (71.3)	69 (62–81)	52 Del19, 35 L858R	2 IIIB, 85 IV	31/10 (46 missing)	86 AD, 1 SCC	NR/NR
Liang SK, 2017	Taiwan	First-line only	140	87 (62.1)	61 (28–87)	81 Del19, 24 L858R, 35 uncommon	4 IIIB, 136 IV	129/11	140 AD	81/59
Liang SK, 2018	Taiwan	First-line only	259	157 (60.6)	62 (28–87)	151 Del19, 53 L858R, 55 uncommon	259 advanced	240/19	259 AD	139/120
Lin YT, 2019	Taiwan	First-line only	99	61 (61.6)	60 (53–71) IQR	59 Del19, 23 L858R, 13 uncommon	99 advanced	92/7	95 AD, 4 non-AD	NR/NR
Liu CY, 2017	Taiwan	First-line only	146	78 (53.4)	63.2 ± 11.3	73 Del19, 61 L858R, 12 uncommon	16 IIIB, 130 IV	123/23	146 AD	79/67
Shen YC, 2017	Taiwan	First-line only	24	15 (62.5)	59 (33–86)	24 uncommon	1 IIIB, 23 IV	19/5	24 AD	24/NR
Sonehara K, 2019	Japan	First-line only	62	36 (58.1)	67 (46–85)	42 Del19, 15 L858R, 3 uncommon	5 I-IIIA, 5 IIIB, 40 IV, 13 recurrence	57/5	61 AD, 1 unclassified	23/39
Tamura K, 2019	Japan	Mixed	1602	947 (59.1)	67 (34–90)	1020 Del19, 421 L858R, 137 uncommon	94 IIIB, 1206 IV, other 301, 1 missing	1381/221	1554 AD, 14 SCC, 2 NOS, 1 large cell, 32 others, 1 unknown	580/1008
Tan WL, 2018	Singapore	First-line only	125	61 (48.8)	62 (26–86)	87 Del19, 27 L858R, 11 uncommon	125 IV	NR	121 AD, 1 SCC, 3 NOS	62/62
Tanaka H, 2019	Japan	First-line only	76	52 (68.4)	68 (42–88)	46 Del19, 28 L858R, 2 uncommon	9 IIIB, 45 IV, 22 recurrence	67/9	74 AD, 1 SCC, 1 NOS	18/58
		Second-line or more	52	41 (78.9)	65 (39–90)	29 Del19, 21 L858R, 2 uncommon	52 recurrence	46/6	51 AD, 1 SCC	NR/NR
Tu CY, 2018	Taiwan	First-line only	104	65 (62.5)	58 < 65 years/46 > 65 years	58 Del19, 23 L858R, 23 uncommon	3 IIIB, 101 IV	93/11	104 AD	67/31
Wada Y, 2016	Japan	Mixed	73	46 (63.0)	69 (42–85)	44 Del19, 20 L858R, 5 uncommon, 4 unknown	1 IIIA, 6 IIIB, 44 IV, 22 recurrence	56/17	75 AD	36/37
Wang S, 2019	China	Mixed	60	30 (50.0)	58.1 (36.3–82.7)	26 Del19, 16 L858R, 18 Uncommon	60 advanced	60/0	60 AD	37/23
		First-line only	39	23 (59.0)	57.2 (36.3–82.7)	19 Del19, 7 L858R, 13 uncommon	39 advanced	39/0	39 AD	NR/NR
		Second-line or more	21	7 (33.3)	59.9 (39.7–75.5)	7 Del19, 9 L858R, 5 uncommon	21 advanced	21/0	21 AD	NR/NR
Yang CJ, 2017	Taiwan	First-line only	48	30 (62.5)	64.6 ± 8.9	29 Del19, 19 L858R	48 IV	38/10	48 AD	19/29

NR, not reported; AD, adenocarcinoma; SCC, squamous cell carcinoma; NOS, not otherwise specified; ECOG-PS, Eastern Cooperative Oncology Group Performance Status; IQR, interquartile range.

**Table 2:** Characteristics of 9 studies in qualitative analysis.

Study	Area	Afatinib	N	Female, *n* (%)	Age, years, median (range)	Mutations	Clinical stage	ECOG-PS, 0–1/≥2	Histology
Fujiwara A 2018	Japan	First-line only	28	19 (67.9)	68 (37–82)	EGFR mutation	8 IIIA, 3 IIIB, 16 IV, 1 recurrence	NR	23 AD, 3 SCC, 2 other
Hochmair MJ, 2018	Global	First-line only	204	110 (53.9)	60 (30–86)	150 Del19, 53 L858R, 1 uncommon	197 IV, 7 missing	153/31 (20 missing)	NR
Jung HA, 2020	Korea	First-line only	61	NR	NR	Uncommon EGFR mutation	NR	NR	NR
Kanazu M, 2020	Japan	Mixed	12	NR	NR	Uncommon EGFR mutation	12 advanced	NR	NR
Kuan FC, 2017	Taiwan	First-line only	81	42 (51.9)	64 (37–83)	48 Del19, 33 L858R	7 IIIB, 74 IV	70/11	81 AD
Lim, J 2019	USA	First-line only	550	355 (64.5)	63.3 ± 11.4	EGFR mutation	NR	NR	NR
Su VY, 2020	Taiwan	First-line only	99	52 (52.5)	64.1 ± 10.8	53 Del19, 31 L858R, 15 uncommon	4 IIIB, 95 IV	89/10	96 AD, 3 non-AD
Wu SG, 2020 (a)	Taiwan	First-line only	36	27 (75)	68.7 (43.0–86.1)	36 uncommon	32 IV, 4 recurrence	32/4	36 AD
Wu SG, 2020 (b)	Taiwan	First-line only	91	44 (48.4)	63 (37–83)	59 Del19, 21 L858R, 11 uncommon	83 advanced, 8 recurrence	NR	91 AD

*Notes*: NR, not reported; AD, adenocarcinoma; SCC, squamous cell carcinoma; ECOG-PS, Eastern Cooperative Oncology Group Performance Status.

**Table 3:** Subgroup analysis based on afatinib medication timing and data sources.

Group	No. of studies	HR (95%CI)	*P*_*A*_	Heterogeneity test	
				*P*_*H*_	I^2^ (%)
**PFS**	8	1.20 (0.95, 1.51)	0.124	0.508	0.0
Management of afatinib					
** **First-line	7	1.23 (0.98, 1.56)	0.080	0.570	0.0
** **≥Second-line	1	0.60 (0.19, 1.87)	0.379	—	—

Calculated HR (95%CI)					
** **Yes	5	1.06 (0.77, 1.44)	0.728	0.483	0.0
** **No	3	1.39 (0.99, 1.96)	0.057	0.486	0.0

*Notes*: *P*_*A*_: *P* value for the test of association; *P*_*H*_: *P* value for the test of heterogeneity.

**Table 4:** Total AEs (or SAEs) based on different afatinib dose (non-full dose vs. full-dose).

AEs (or SAEs)	Non-full dose		Full dose	
	Total	Grade ≥3	Total	Grade ≥3
Diarrhea	55.8 (41.1, 70.0)%	1.0 (0.0, 7.4)%	94.7 (86.8, 99.5)%	9.8 (3.0, 19.3) %
Skin rashes	77.7 (64.2, 88.9)%	0.0 (0.0, 4.0) %	90.3 (80.9, 97.1)%	7.7 (1.7, 16.5) %
Mucositis	34.7 (21.4, 49.2)%	0.0 (0.0, 4.0) %	58.0 (44.9, 70.6)%	0.0 (0.0, 3.0) %
Paronychia	48.9 (34.4, 63.5)%	1.5 (0.0, 8.4) %	74.0 (61.7, 84.7) %	7.7 (1.7, 16.5) %
Dry skin	55.6 (40.9, 69.8)%	0.0 (0.0, 4.0) %	51.0 (38.0, 64.0) %	0.0 (0.0, 3.0) %
Pruritus	16.7 (6.9, 29.3)%	0.0 (0.0, 4.0) %	21.5 (11.6, 33.3) %	0.0 (0.0, 3.0) %

AEs, adverse events; SAEs, severe adverse events.

## Data Availability

The raw data supporting the conclusions of this manuscript will be made available by the authors, without undue reservation, to any qualified researcher.

## Abbreviations

CI: Confidence interval
DCR: Disease control rate
ECOG-PS: Eastern Cooperative Oncology Group Performance Status
EGFR: Epidermal growth factor receptor
HR: Hazard risk
IR: Incidence rate
NSCLC: Non-small-cell lung cancer
ORR: Overall response rate
OS: Overall survival
PFS: Progression-free survival
RCT: Randomized clinical trial
RR: Relative risk
RWE: Real-world evidence
TTF: Time to failure
TKIs: Tyrosine kinase inhibitors
WMD: Weighted mean difference
AE: Adverse event
SAE: Severe adverse event.

## Supplementary Materials

Supplementary Materials Supplementary Figure 1: the meta-analysis results for various incidence rates of adverse events after afatinib treatment in advanced NSCLC with EGFR mutation: (A) the incidence rate of fatigue; (B) the incidence rate of increased alanine aminotransferase (ALT); (C) the incidence rate of increased aspartate aminotransferase (AST) levels; and (D) the incidence rate of interstitial lung disease (ILD). Supplementary Figure 2: the meta-analysis results for incidence rates of severe adverse events after afatinib treatment in advanced NSCLC with EGFR mutation: (A) the adverse reaction incidence rate of fatigue; (B) the adverse reaction incidence rate of increased alanine aminotransferase (ALT) levels; (C) the adverse reaction incidence rate of increased aspartate aminotransferase (AST) levels; and (D) the adverse reaction incidence rate of interstitial lung disease (ILD). Supplementary Figure 3: the meta-analysis results for risk of progression-free survival (PFS) after afatinib treatment in advanced NSCLC with EGFR mutation: (A) PFS between brain metastases group vs. non-brain metastases group and (B) PFS between exon 19 deletion *vs*. uncommon, exon 19 deletion *vs*. exon 21 L858R, brain metastases (no vs. yes), and ECOG-PS (0–1) vs. ECOG-PS (≥2).
