# Supplementary figures and images for: Efficacy and Safety of Afatinib in the Treatment of Advanced Non-Small-Cell Lung Cancer with EGFR Mutations: A Meta-Analysis of Real-World Evidence
Source: J Oncol. 2021 Dec 18;2021:8736288. doi: 10.1155/2021/8736288 (PMC8710163; doi:10.1155/2021/8736288)

A

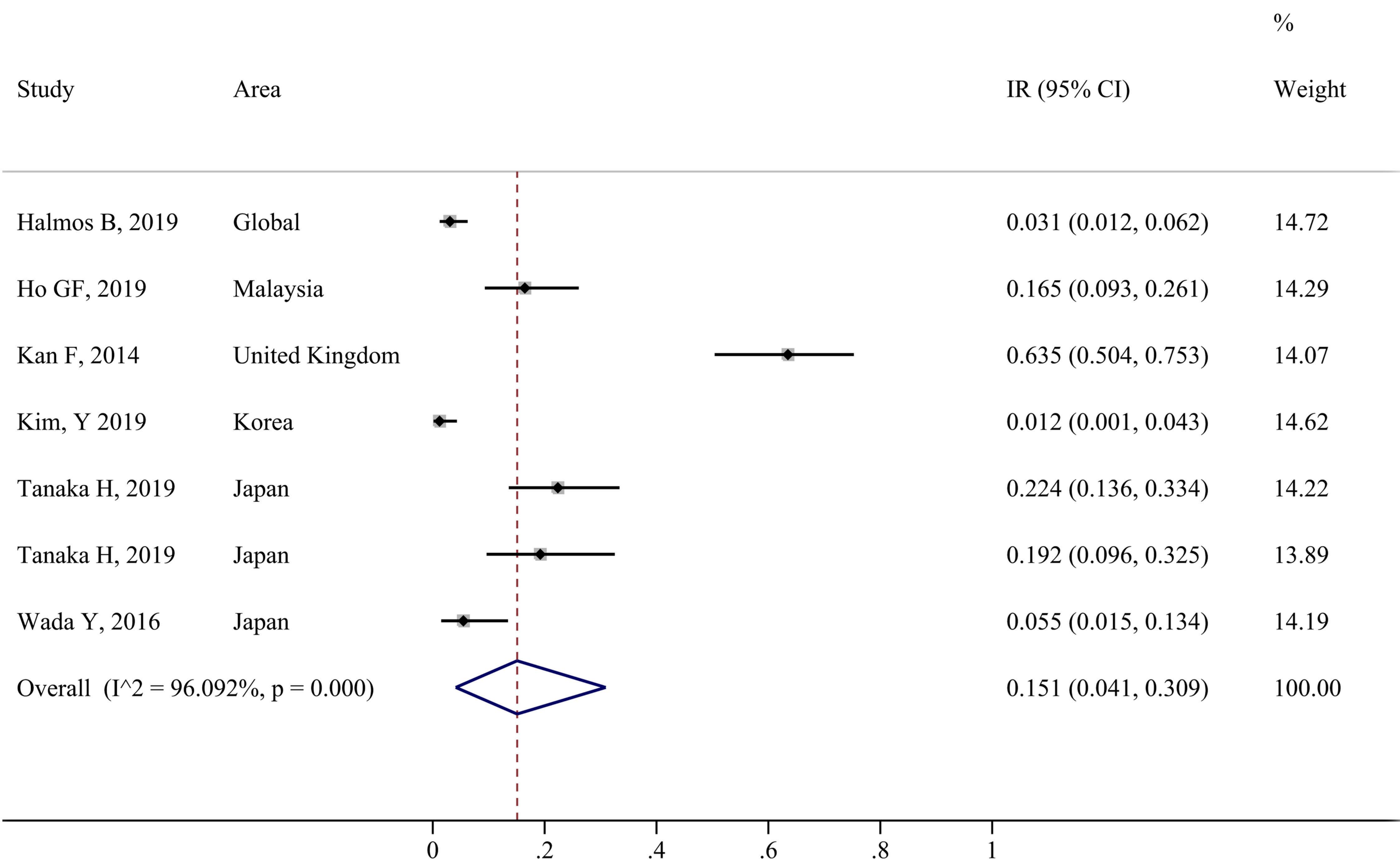

B

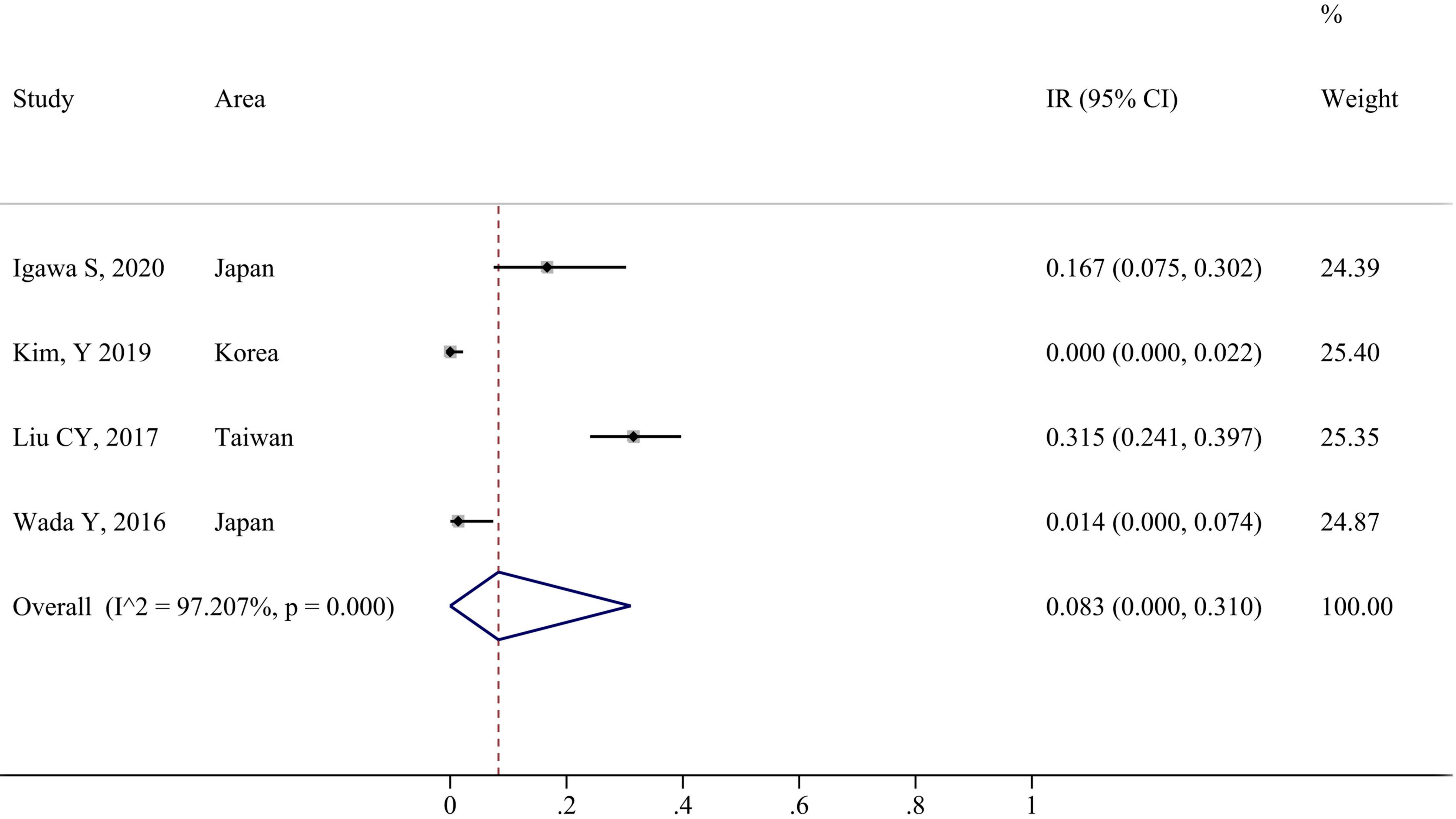

C

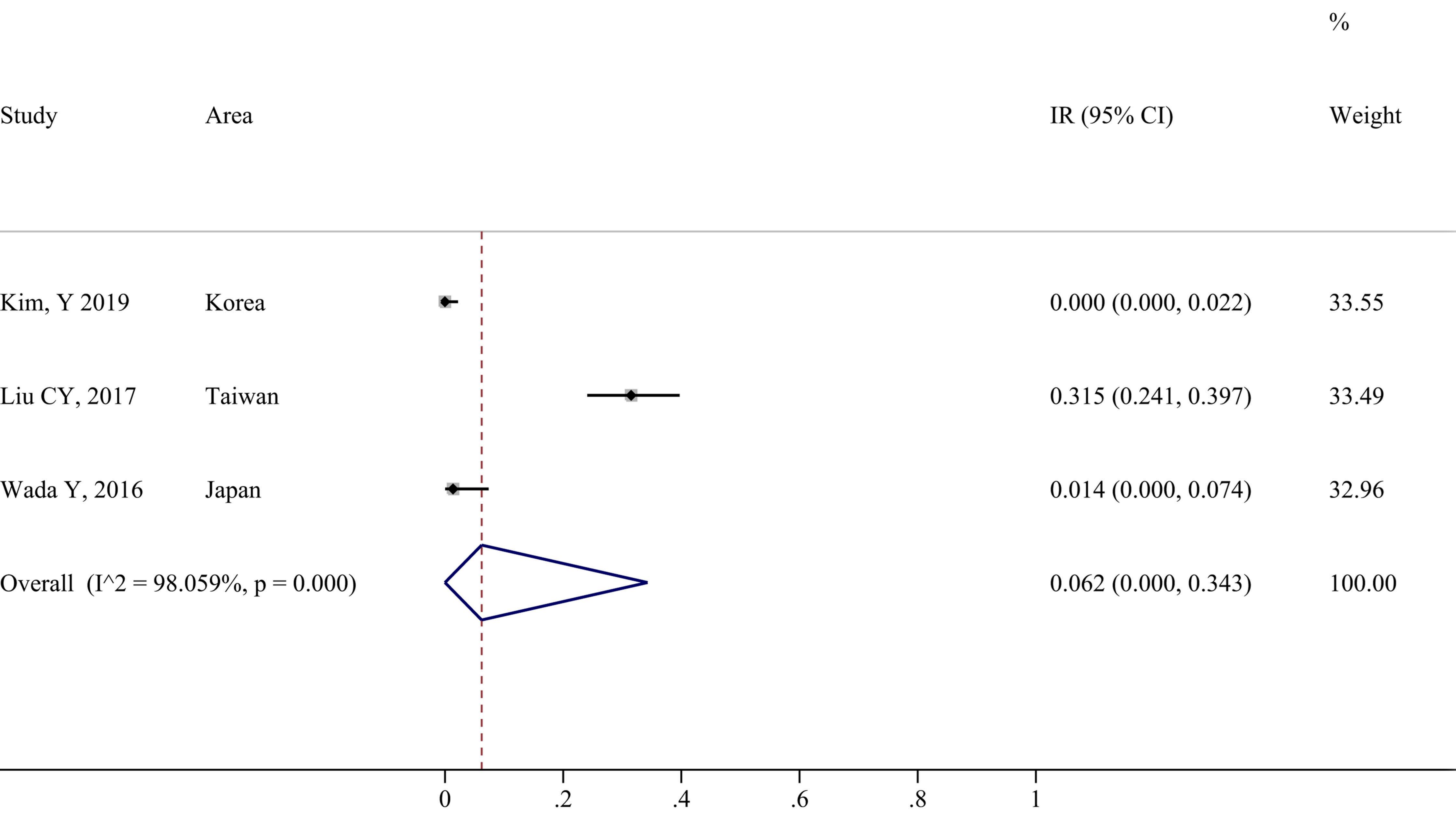

D

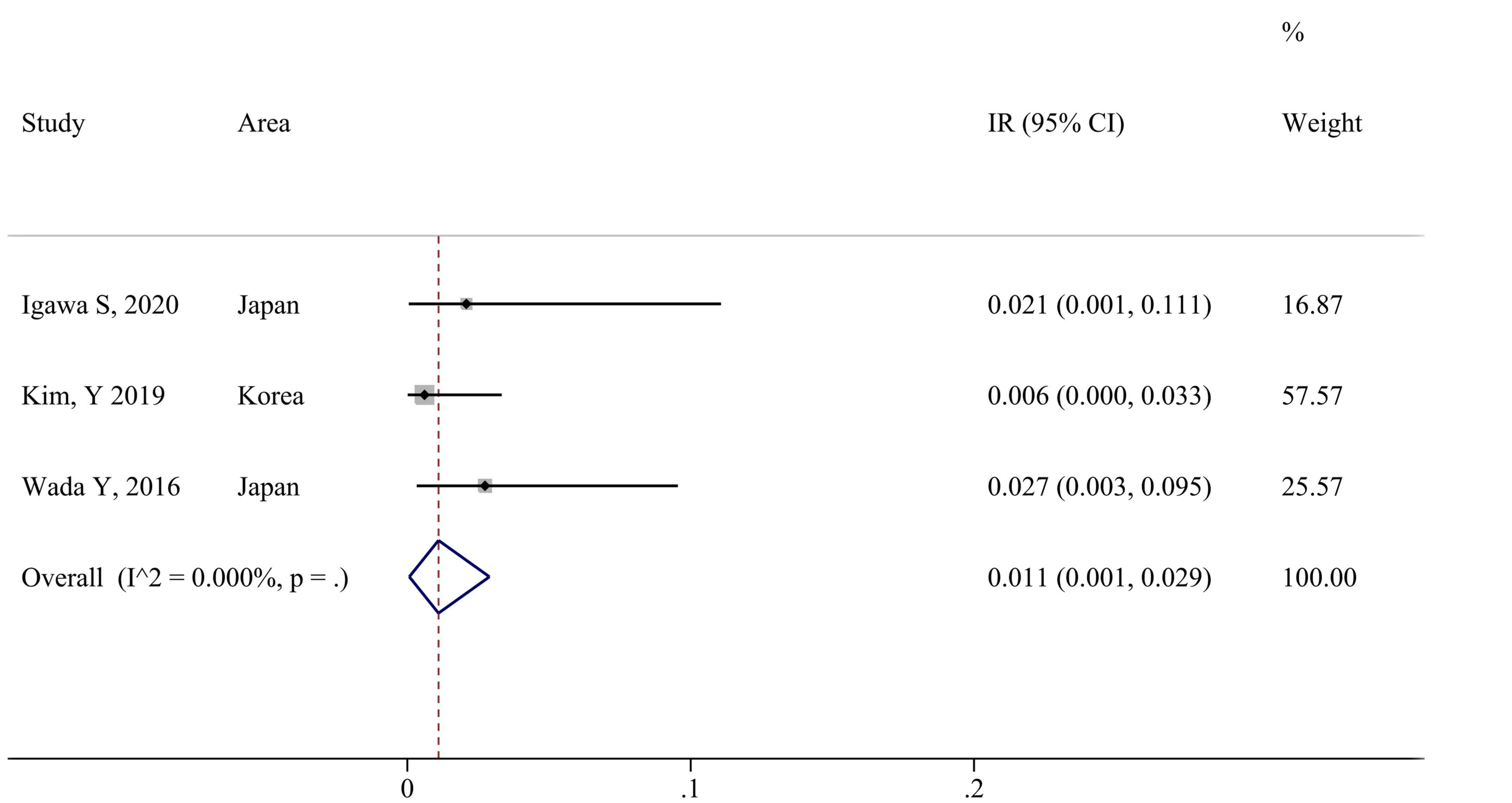

Supplement: Supplementary Materials — Supplementary Figure 1: the meta-analysis results for various incidence rates of adverse events after afatinib treatment in advanced NSCLC with EGFR mutation: (A) the incidence rate of fatigue; (B) the incidence rate of increased alanine aminotransferase (ALT); (C) the incidence rate of increased aspartate aminotransferase (AST) levels; and (D) the incidence rate of interstitial lung disease (ILD). Supplementary Figure 2: the meta-analysis results for incidence rates of severe adverse events after afatinib treatment in advanced NSCLC with EGFR mutation: (A) the adverse reaction incidence rate of fatigue; (B) the adverse reaction incidence rate of increased alanine aminotransferase (ALT) levels; (C) the adverse reaction incidence rate of increased aspartate aminotransferase (AST) levels; and (D) the adverse reaction incidence rate of interstitial lung disease (ILD). Supplementary Figure 3: the meta-analysis results for risk of progression-free survival (PFS) after afatinib treatment in advanced NSCLC with EGFR mutation: (A) PFS between brain metastases group vs. non-brain metastases group and (B) PFS between exon 19 deletion vs. uncommon, exon 19 deletion vs. exon 21 L858R, brain metastases (no vs. yes), and ECOG-PS (0–1) vs. ECOG-PS (≥2). [file 8736288.f1.zip › 8736288.f1/Supplementary Figure 1.pdf]

A

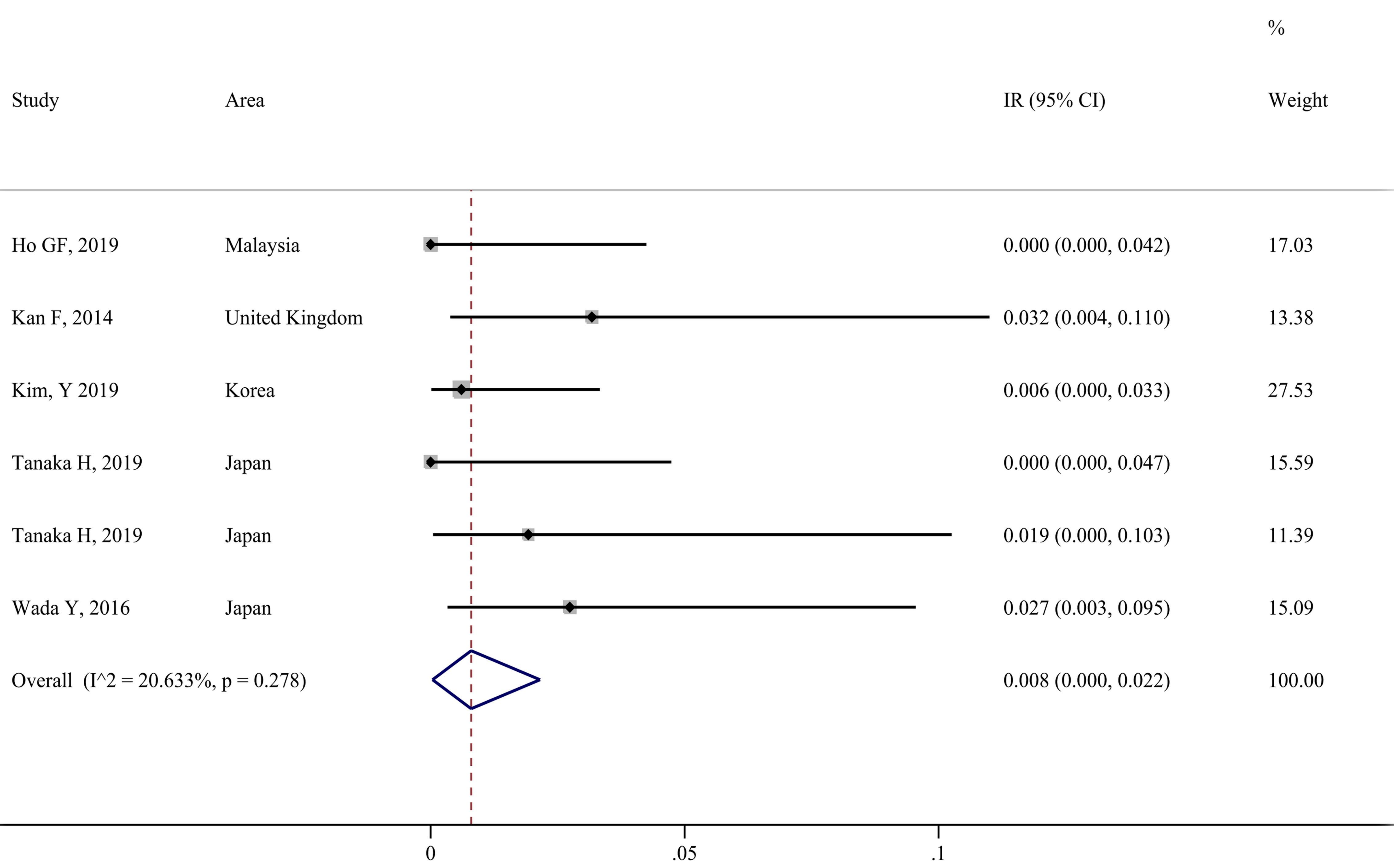

B

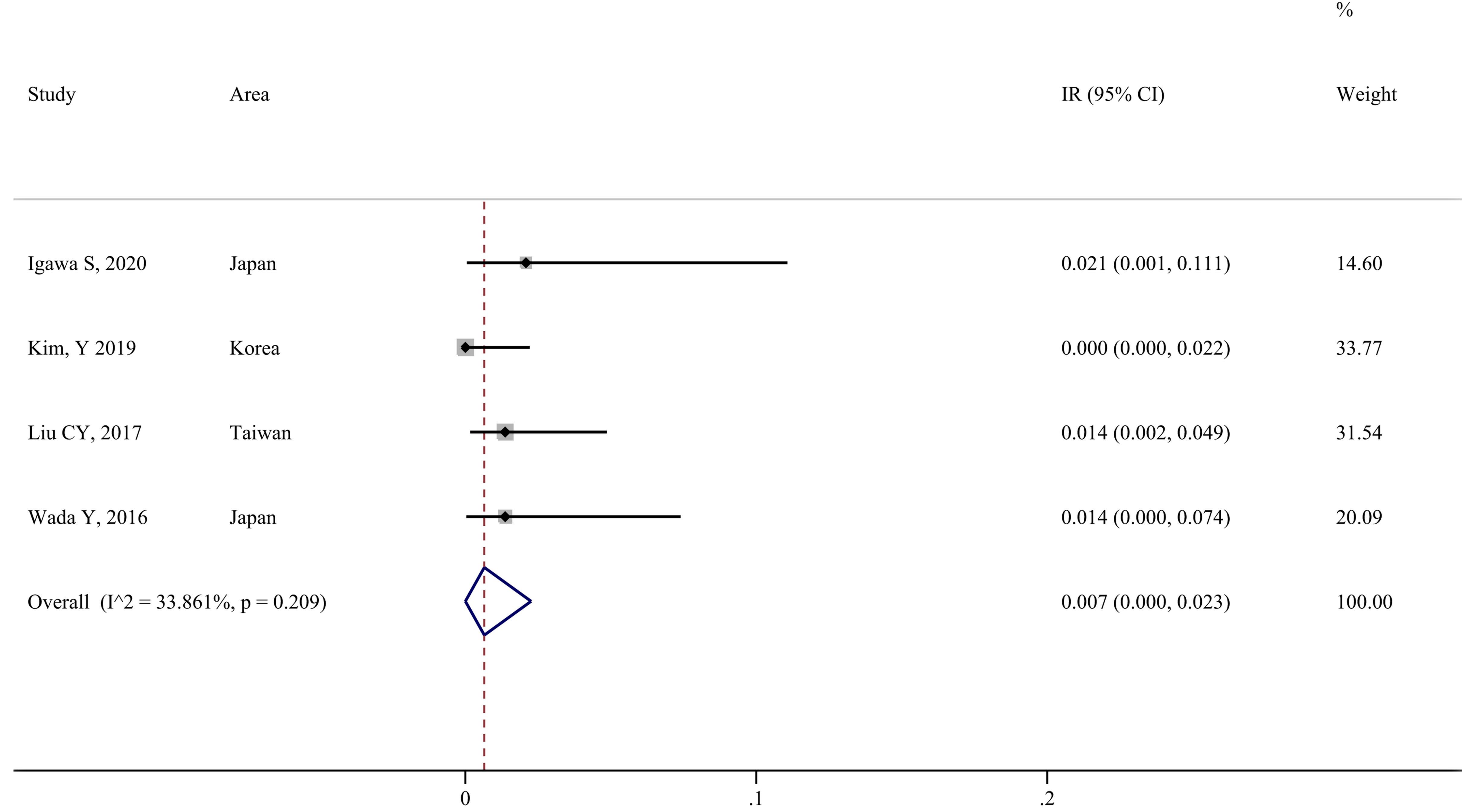

C

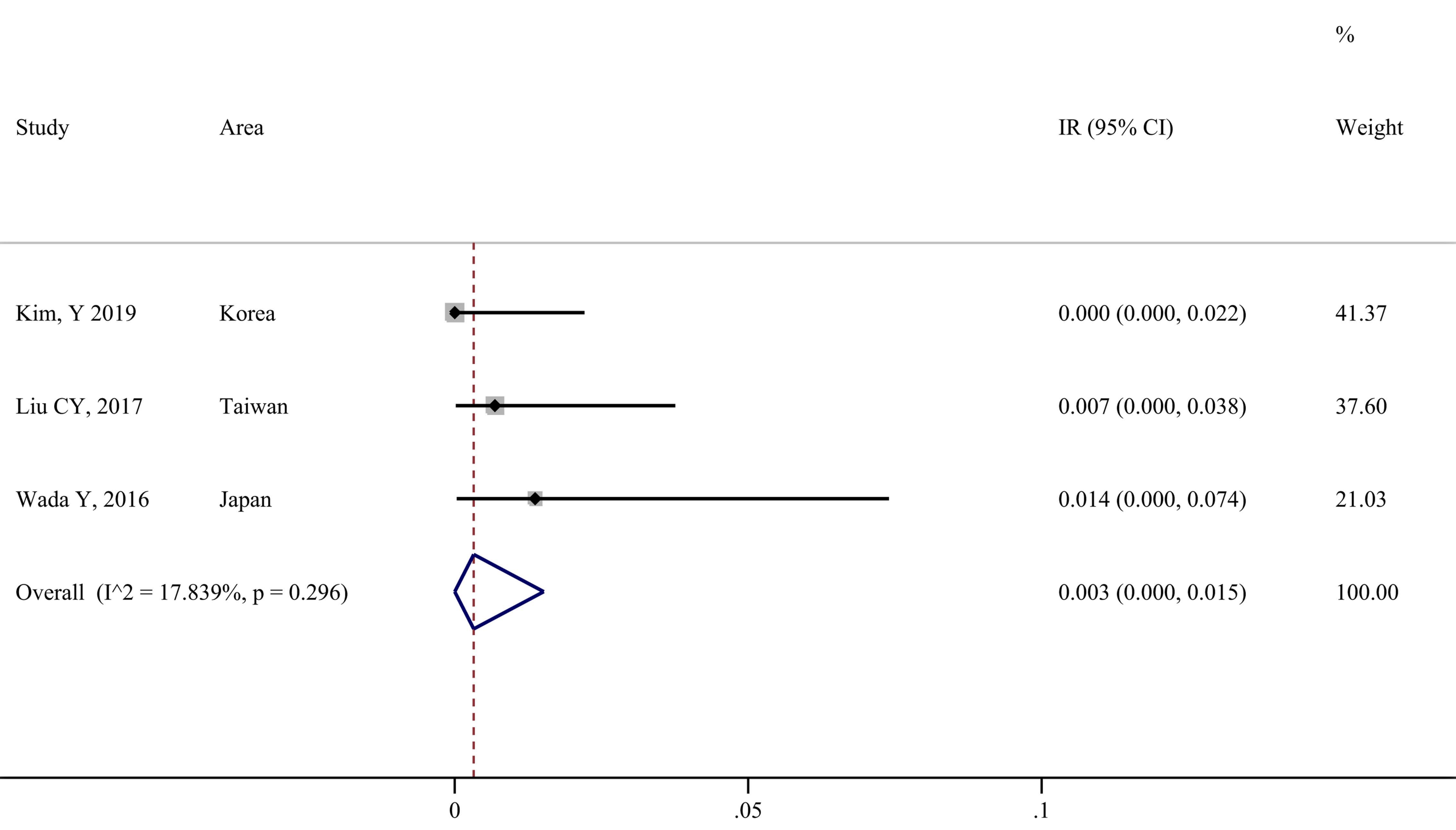

D

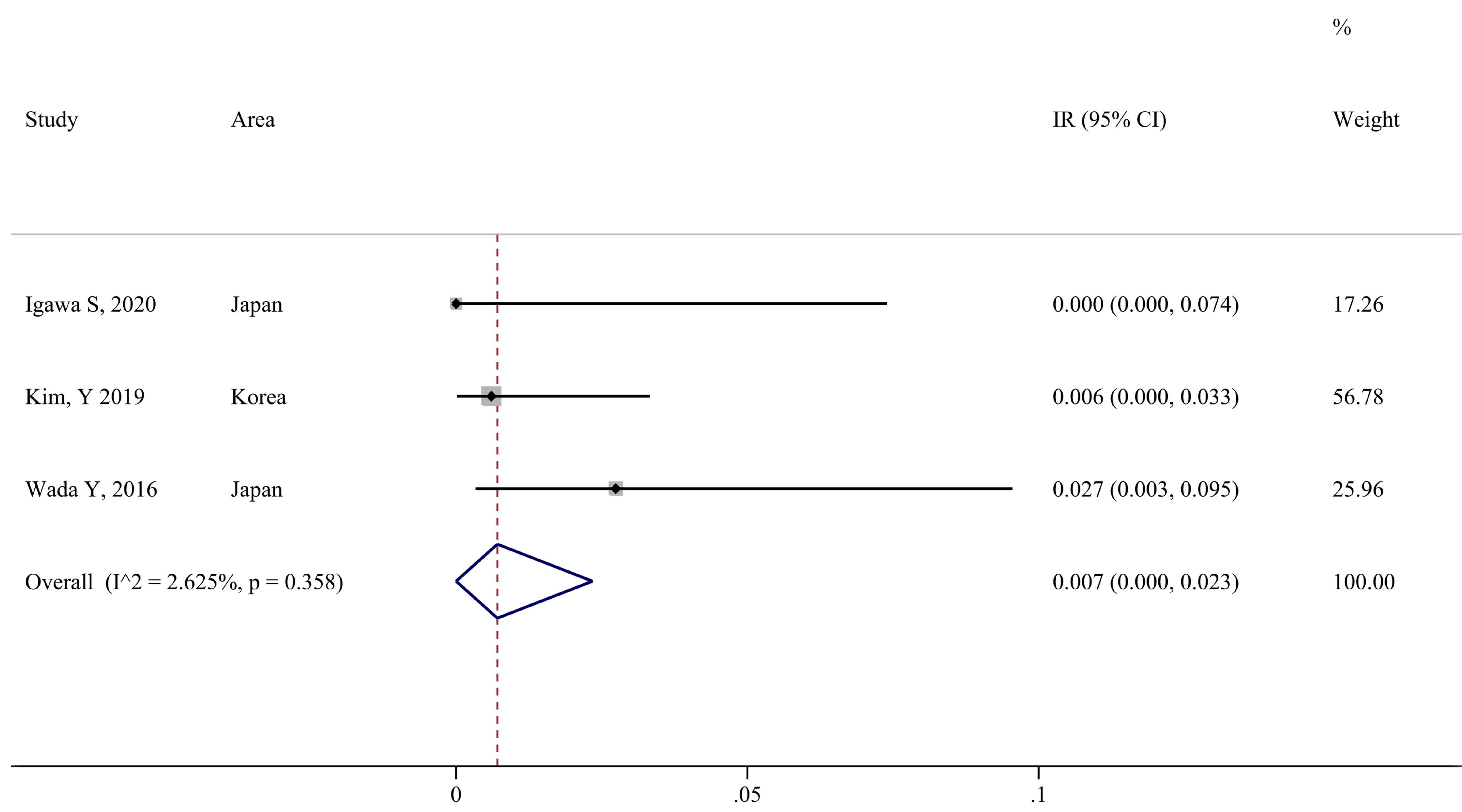

Supplement: Supplementary Materials — Supplementary Figure 1: the meta-analysis results for various incidence rates of adverse events after afatinib treatment in advanced NSCLC with EGFR mutation: (A) the incidence rate of fatigue; (B) the incidence rate of increased alanine aminotransferase (ALT); (C) the incidence rate of increased aspartate aminotransferase (AST) levels; and (D) the incidence rate of interstitial lung disease (ILD). Supplementary Figure 2: the meta-analysis results for incidence rates of severe adverse events after afatinib treatment in advanced NSCLC with EGFR mutation: (A) the adverse reaction incidence rate of fatigue; (B) the adverse reaction incidence rate of increased alanine aminotransferase (ALT) levels; (C) the adverse reaction incidence rate of increased aspartate aminotransferase (AST) levels; and (D) the adverse reaction incidence rate of interstitial lung disease (ILD). Supplementary Figure 3: the meta-analysis results for risk of progression-free survival (PFS) after afatinib treatment in advanced NSCLC with EGFR mutation: (A) PFS between brain metastases group vs. non-brain metastases group and (B) PFS between exon 19 deletion vs. uncommon, exon 19 deletion vs. exon 21 L858R, brain metastases (no vs. yes), and ECOG-PS (0–1) vs. ECOG-PS (≥2). [file 8736288.f1.zip › 8736288.f1/Supplementary Figure 2.pdf]

A

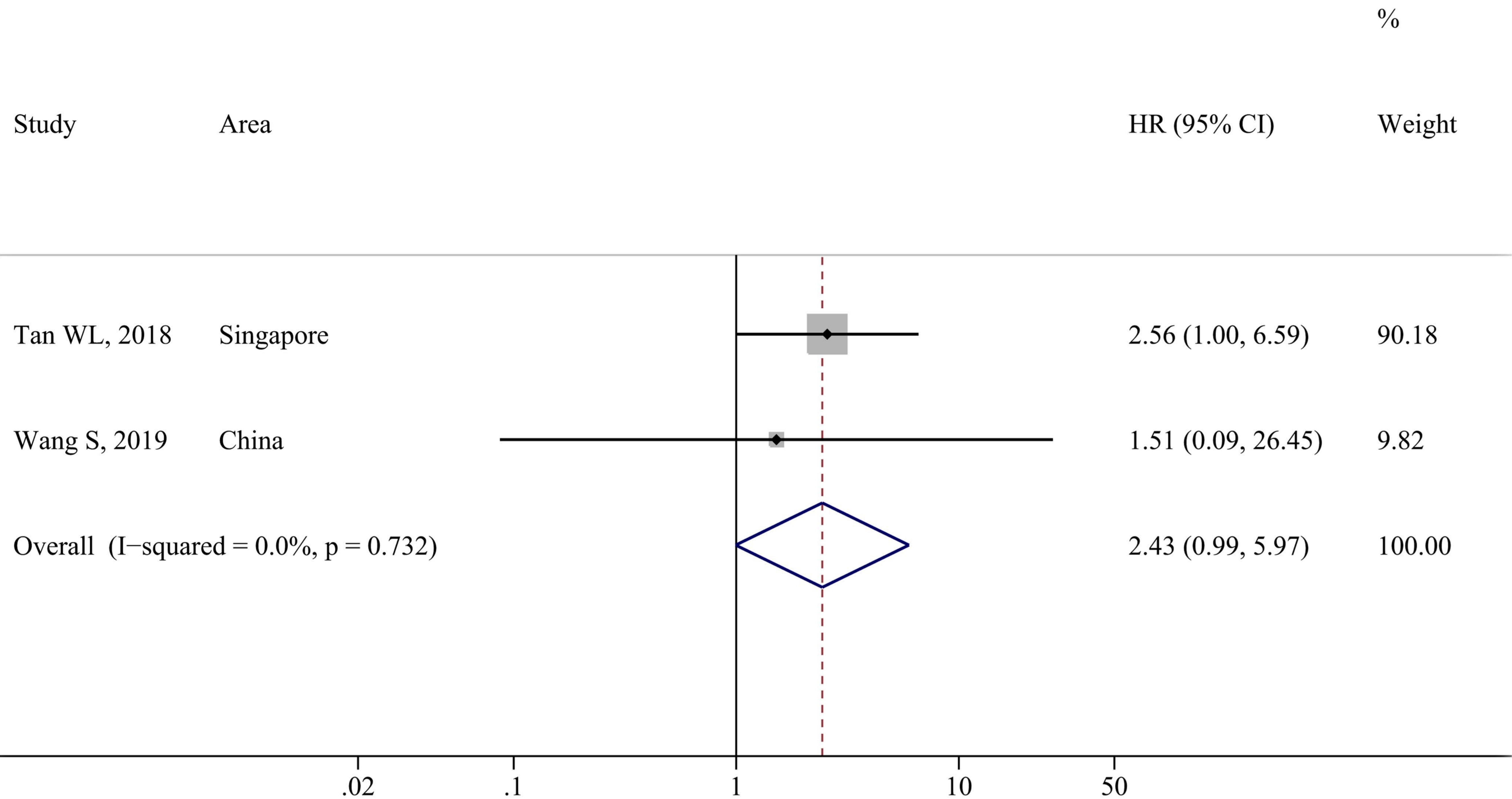

B

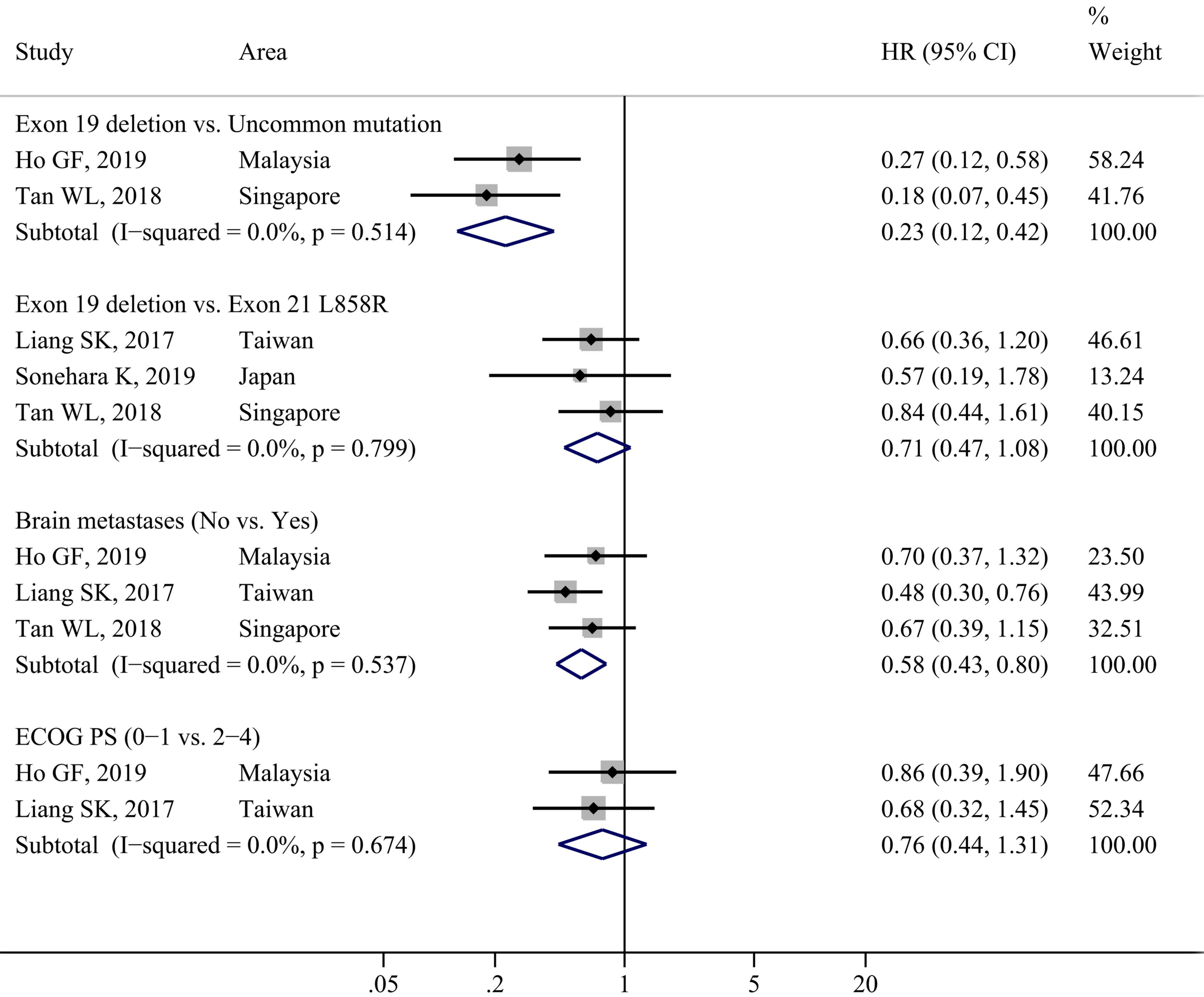

Supplement: Supplementary Materials — Supplementary Figure 1: the meta-analysis results for various incidence rates of adverse events after afatinib treatment in advanced NSCLC with EGFR mutation: (A) the incidence rate of fatigue; (B) the incidence rate of increased alanine aminotransferase (ALT); (C) the incidence rate of increased aspartate aminotransferase (AST) levels; and (D) the incidence rate of interstitial lung disease (ILD). Supplementary Figure 2: the meta-analysis results for incidence rates of severe adverse events after afatinib treatment in advanced NSCLC with EGFR mutation: (A) the adverse reaction incidence rate of fatigue; (B) the adverse reaction incidence rate of increased alanine aminotransferase (ALT) levels; (C) the adverse reaction incidence rate of increased aspartate aminotransferase (AST) levels; and (D) the adverse reaction incidence rate of interstitial lung disease (ILD). Supplementary Figure 3: the meta-analysis results for risk of progression-free survival (PFS) after afatinib treatment in advanced NSCLC with EGFR mutation: (A) PFS between brain metastases group vs. non-brain metastases group and (B) PFS between exon 19 deletion vs. uncommon, exon 19 deletion vs. exon 21 L858R, brain metastases (no vs. yes), and ECOG-PS (0–1) vs. ECOG-PS (≥2). [file 8736288.f1.zip › 8736288.f1/Supplementary Figure 3.pdf]
